# Effect of Heating Temperature of High-Quality Arbequina, Picual, Manzanilla and Cornicabra Olive Oils on Changes in Nutritional Indices of Lipid, Tocopherol Content and Triacylglycerol Polymerization Process

**DOI:** 10.3390/molecules28104247

**Published:** 2023-05-22

**Authors:** Dominik Kmiecik, Monika Fedko, Justyna Małecka, Aleksander Siger, Przemysław Łukasz Kowalczewski

**Affiliations:** 1 Department of Food Technology of Plant Origin, Poznań University of Life Sciences, 31 Wojska Polskiego St., 60-624 Poznań, Poland; przemyslaw.kowalczewski@up.poznan.pl; 2 Division of Fat and Oils and Food Concentrates Technology, Department of Food Technology and Assessment, Institute of Food Science, Warsaw University of Life Sciences, 159c Nowoursynowska St., 02-787 Warsaw, Poland; monika_fedko@sggw.edu.pl; 3 Liberado Justyna Małecka Oliwny Raj, 233 Dąbrowskiego St., 60-406 Poznań, Poland; justyna@liberado.pl; 4 Department of Food Biochemistry and Analysis, Poznań University of Life Sciences, 31 Wojska Polskiego St., 60-634 Poznań, Poland; aleksander.siger@up.poznan.pl

**Keywords:** extra virgin olive oils, lipid nutritional quality, thermal stability, heating process, thermal degradation

## Abstract

The aim of the study was to determine the stability and heat resistance of extra premium olive oil. The study material consisted of six extra virgin olive oils (EVOO) obtained from Spain. Four samples were single-strain olive oils: Arbequina, Picual, Manzanilla, and Cornicabra. Two samples were a coupage of Arbequina and Picual varieties: Armonia (70% Arbequina and 30% Picual) and Sensation (70% Picual and 30% Arbequina). Olive oil samples were heated at 170 °C and 200 °C in a pan (thin layer model). In all samples, changes in indexes of lipid nutritional quality (PUFA/SFA, index of atherogenicity, index of thrombogenicity, and hypocholesterolemic/hypercholesterolemic ratio), changes in tocopherol, total polar compounds content, and triacylglycerol polymers were determined. Heating olive oil in a thin layer led to its degradation and depended on the temperature and the type of olive oil. Increasing the temperature from 170 to 200 °C resulted in significantly higher degradation of olive oil. At 200 °C, deterioration of lipid nutritional indices, total tocopherol degradation, and formation of triacylglycerol polymers were observed. A twofold increase in the polar fraction was also observed compared to samples heated at 170 °C. The most stable olive oils were Cornicabra and Picual.

## 1. Introduction

The olive tree (*Olea europaea* L.) is common in Mediterranean countries. Olive trees are one of the first tree species to be cultivated and, according to sources, the first records go back 6000 years [1,2]. The main producers of olives are still the Mediterranean countries, mainly Spain, Italy, and Greece, which makes the European Union their global producer, responsible for 70% of the global production of olives [3]. The most valuable part of olive tree is its fruits, and, as a result of pressing their pulp, olive oil is obtained [1]. Growing olive trees, harvesting olives, and then extracting the liquid to produce olive oil are inseparable from the history and culture of the Mediterranean people. The great importance of olive trees and olive oil in the development of civilization is confirmed by the recognition of them as sacred in the three largest religions (Judaism, Christianity, and Islam) [4]. The influence of olive oil on health has been described in history for thousands of years, and the first reports date back to the times of the great Greek philosophers, both Aristotle and Hippocrates as well [5]. Olive oil was used not only as a source of food, but also as fuel for lamps, lubricants for athletes and warriors, and in religious rituals [4]. However, ongoing scientific research confirms the wisdom of the ancients in the context of the beneficial properties of olive oil for our health [6].

Extra virgin olive oil (EVOO) is chemically composed mainly of triacylglycerols and contains small amounts of free fatty acids, glycerol, phosphatides, colorants, flavor compounds, and sterols. It also contains many antioxidant compounds, mainly tyrosol and hydroxytyrosol and their derivatives, lignans pinoresinol, 1-acetoxypinoresinol, luteolin, apigenin, and phenolic acids [7,8]. In addition, other polyphenolic compounds in EVOO are tocopherols, tocotrienols, triterpenoid derivatives, ursolic acid, uvaol, and oleanolic acid; *o*-diphenols; flavonoid polyphenols such as quercetin, luteolin, and rutin; and pigments (chlorophyll, carotenoids) [4,9,10]. 

**Figure 1:**
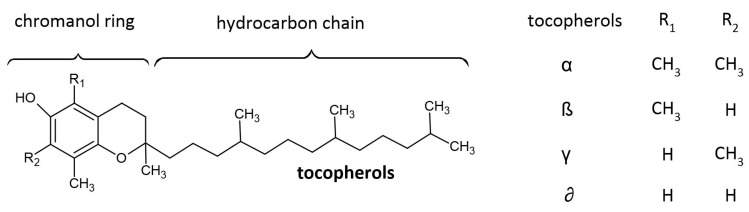
Structure of the main tocopherols present in olive oils.

Tocopherols are among the most important native antioxidants observed in oils. Their content varies widely and depends on the raw material, as well as the conditions of cultivation and extraction of the oil. A rich source of tocopherols is wheat germ oil (210 mg/100 g of oil) and also soybean oil (105 mg/100 g of oil), corn oil (77 mg/100 g of oil), or rapeseed oil (80 mg/100 g of oil) [11]. Tocopherols can be found in four basic forms α, β, λ, and γ. In olive oil, the main tocopherol is α-tocopherol, which accounts for more than 95% of total tocopherols [12,13]. The structure of the main tocopherols is presented in Figure 1.

The fats present in food are made up of fatty acids, which we can divide into saturated fatty acids (SFA), monounsaturated fatty acids (MUFA), and polyunsaturated fatty acids (PUFA). The different groups of fatty acids affect our health in different ways. SFA are seen as those that, in excessive amounts, cause our health to deteriorate. MUFA and PUFA belong to the group defined as health-promoting. In the case of glyceride fraction, EVOO has a high fatty acid content and, in particular, a high proportion of monounsaturated fatty acids (MUFA), mainly oleic acid (C 18:1) and palmitoleic acid (C 16:1) but contains also polyunsaturated fatty acids (PUFA) such as linoleic (C 18:2). Saturated fatty acids (SFA) account for about 14% of total fat, with palmitic and stearic acids being the main components [14,15]. The fatty acid composition is characteristic of the fruit variety from which the oil is pressed [16]. In addition to olive variety, fatty acid composition is affected by the maturity index and region and growing conditions [17]. A high concentration of MUFA in the diet increases HDL cholesterol levels and reduces triglycerides. In addition, EVOO improves postprandial lipemia, and these effects appear to be particularly beneficial for patients with type 2 diabetes [18,19].

Olive oil has a wide range of uses in food production, especially in the Mediterranean diet. It is used as an ingredient in dressings, for cooking and frying, and even as a substitute for animal fats in meat products [20,21,22]. Due to the high content of oleic acid and significant amounts of antioxidant compounds, olive oil can be successfully used in food preparation, including thermal processes [23,24]. The use of the proper temperature during frying will not only allow obtaining tastier dishes, but above all, it will protect against negative oxidative changes of fatty acids [25]. In addition, the quality of olive oil is important and is determined by the maturity of the olives from which the oil is obtained, climatic conditions at the time of cultivation, and even the variety [26]. 

**Figure 2:**
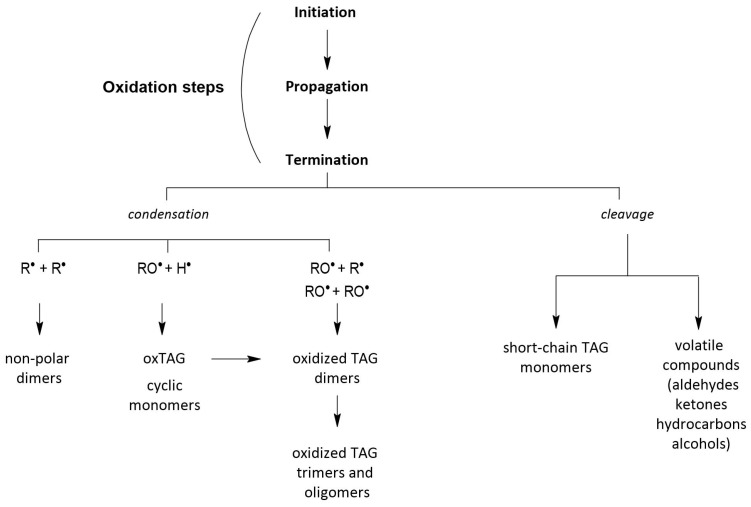
Scheme of oxidative transformations of fats during heat treatment.

When oils are used in the frying process, a number of undesirable changes in their quality and structure are observed. As a result of high temperature, oxygen from air, and water from the fried product, rapid processes of fat oxidation, hydrolysis, and polymerization of triacylglycerols occur. During these reactions, compounds of lower and higher molecular weight relative to native triacylglycerols are formed (Figure 2).

**Figure 3:**
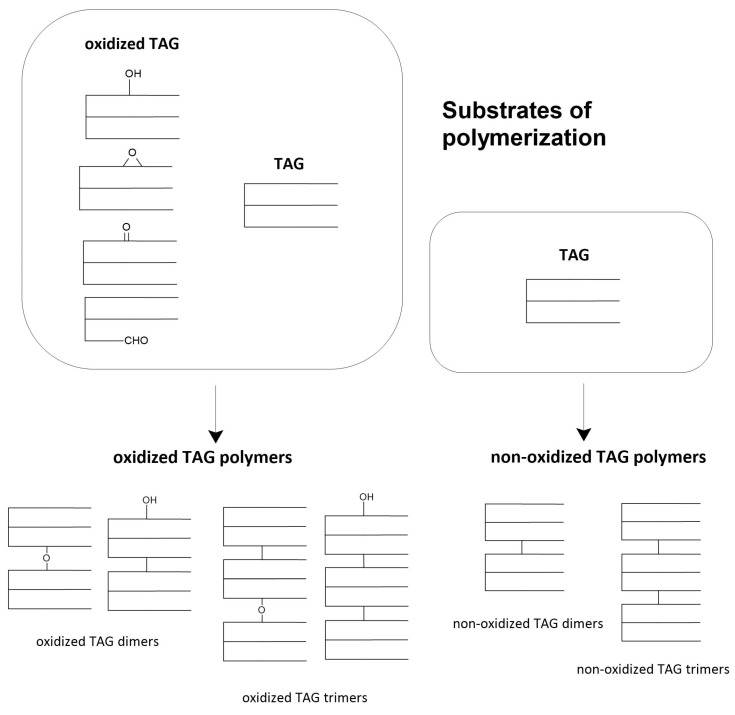
Scheme of the substrates and products of the triacylglycerol polymerization process.

In the first group, we can include volatile compounds, hydrocarbons, free fatty acids, alcohols, etc. In the second group, we will observe the products of the polymerization process, dimers, trimers, and oligomers of triacylglycerols (TAG) [27]. The rate of oil degradation during frying depends on the type of oil, heating time, or process temperature. Polymerization of triacylglycerols leads to the formation of non-oxidized and oxidized polymers. The scheme of formation of triacylglycerol polymers is presented in Figure 3.

Oxidation and polymerization of triacylglycerols is an undesirable phenomenon. On the one hand, it negatively affects nutritional values of oils through the formation of oxidized derivatives, lowering the content of vitamins and antioxidants, as well as reducing the nutritional value of ω3 and ω6 polyunsaturated fatty acids. On the other hand, the TAG polymers formed negatively affect the frying process by leading to its prolongation, hindering heat transfer to the product, and increasing the fat content of the fried product [28]. One of the most important factors affecting the speed of the processes that occur when oils are heating is temperature. Frying is a process in which oils are usually heated at temperatures of 170 °C and above. However, often due to lack of control, oils can be used at higher temperatures. Increasing the temperature causes a significant increase in oil degradation processes. In particular, in terms of the reduction of polyunsaturated fatty acids, phytosterol, and tocopherol content and the intensification of thermal transformations leading to cyclization and polymerization of triacylglycerols [29,30,31,32]. An increase in TAG polymers in the human diet may increase oxidative stress in the gut, might negatively affect lipid blood profile, renal functions, or peroxidation of lipids in the human body [33,34]

The polyphenolic compounds present in oils have a documented health-promoting effect in our body [21,35]. They can also help stabilize unsaturated fatty acids during storage and thermal processing [36,37]. EVOO is characterized by a high content of phenolic compounds and squalene. However, their amount depends on both the place and conditions of cultivation and the variety [38]. The variety of fruit used in olive oil production significantly affects the squalene content. Large varietal differences can be seen especially between the Picual and Arbequina varieties. Olive oil from Picual varieties has two to three times lower Squalene content compared to oil obtained from the Arbequina variety [38,39]. However, the Picual variety, as well as Manzanilla, had a 1.5 higher content of phenolic compounds than Arbequina [40]. Such large differences in the content of active compounds can affect the thermal stability of olive oils obtained from different varieties.

Most studies on the stability of olive oil under thermal processes mainly focus on the transformation of bioactive compounds or the stability of the oil as measured by the increment of primary and secondary fatty acid oxidation products. There are much fewer data on the total degradation processes of olive oil expressed by the content of polar compounds and the polymerization process of triacylglycerols. Bearing the aforementioned in mind, it seems crucial to analyze the impact of EVOO quality on the course of changes during its heating. For this purpose, the profile of fatty acids, the content of tocochromanols, chlorophyll, as well as polymerized triacylglycerols, and total polar compounds after a model heating process were analyzed.

## 2. Results and Discussion

### 2.1. Characterization of Fresh Olive Oils

The characteristics of non-heated olive oils are shown in Table 1. All olive oils had similar fatty acid profiles. The main fatty acids were oleic, palmitic, linoleic, and linolenic acid [16,17]. The dominant group of fatty acids in all olives was monounsaturated fatty acids (MUFA). Their proportion ranged from 71.17% (Arbequina) to 80.32% (Picual). Oleic acid was the dominant fatty acid. Polyunsaturated fatty acids (PUFA) accounted for 3.95% (Picual) to 10.53% (Arbequina). Among PUFAs, ω6 linoleic acid was the dominant one. Its proportion ranged from 3.12% to 9.89% and depended on the olive variety. Its content increased in the following order: Picual, Cornicabra, Manzanilla, Sensation, Armonia, and Arbequina. The second of the essential PUFA acids, ω3 linolenic acid, accounted for between 0.63% (Armonia) and 0.85% (Cornicabra) of the total fatty acid. The increase in its proportion was in the order of: Armonia, Arbequina, Manzanilla, Sensation, Picual, and Cornicabra. The mutually appropriate ratio of these acids (ω6/ω3 ratio) may determine the ability to reduce the risk of many chronic diseases. The health-promoting ω6/ω3 ratio is 1:1 to 5:1 [41]. This value was characteristic of four olive oils: Picual, Manzanilla, Cornicabra, and Sensation. In Arbequina olive oil and a blend prepared with its predominant proportion: Armonia, the ω6/ω3 ratio was 15.48 and 14.14, respectively.

In the olive oils tested, the content of tocopherols ranged from 22.49 to 36.16 mg/1 g of oils. The lowest content of tocopherols was observed in olive oil from the Manzanilla variety and the highest in Cornicabra olive. The main tocopherol in olive oils was α-tocopherol, which accounted for 95.3% (Manzanilla) to 97.8% (Arbequina) of all tocopherols. Other tocopherols averaged between 1.2 and 2.1% for β-tocopherol and γ-tocopherol, respectively. However, their content varied and depended on the olive oil variety. The highest amount of β-tocopherol (0.5 mg/1 g of oils) was observed in Cornicabra olive oil. This represented three times more than in Arbequina olive oil and two times more than in Armonia and Picual olive oils. The γ-tocopherol content ranged from 0.47 (Arbequina) to 0.82 (Cornicabra) mg/1 g of oils.

The tested oils also differed when comparing chlorophyll and polar compound content. The chlorophyll content is related to the color of the olive oil obtained. The higher variance was characteristic of darker colored oil. Chlorophyll content, expressed as mg phenophytin/1 kg of oil ranging from 9.51 to 20.27 for Manzanilla and Picual oils, respectively. The high content of chlorophyll in Arbequina and Picual oils influenced its high level in the resulting Armonia and Sensation blends.

The content of the polar fraction in the analyzed oils ranged from 2.55 (Sensation) to 5.54% (Manzanilla). Its content increased in the following order: Sensation, Armonia, Cornicabra, Arbequina, Picual, and Manzanilla. The level of the polar fraction in olive oil depends on the variety, the growing area, the degree of maturity, as well as the oil extraction technique [42].

### 2.2. Indexes of Lipid Nutritional Quality

Vegetable oils are a product that is a rich source of unsaturated fatty acids. However, the ratio between the different groups of fatty acids determines their nutritional quality. To better determine the nutritional quality of vegetable and animal fats, indicators have been determined that characterize their pro-health or pro-inflammatory effects on health [43].

**Figure 4:**
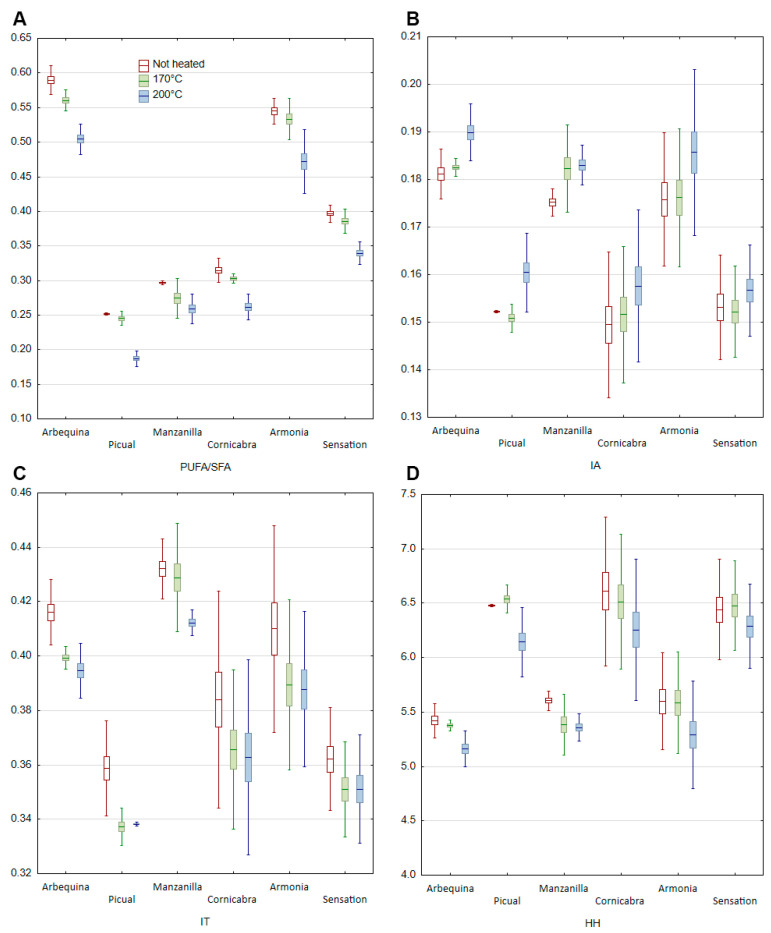
Changes of indexes of lipid nutritional quality of not heated and heated olive oil at 170 °C and 200 °C. (**A**) PUFA/SFA—polyunsaturated and saturated fatty acid ratio, (**B**) IA—index of atherogenicity, (**C**) IT—index of thrombogenicity, (**D**) HH—hypocholesterolemic/hypercholesterolemic ratio.

In unheated olive oil and heated samples based on fatty acid composition, four indicators of nutritional quality were determined: PUFA/SFA—polyunsaturated and saturated fatty acid ratio, IA—index of atherogenicity, IT—index of thrombogenicity, HH—hypocholesterolemic/hypercholesterolemic ratio (Figure 4).

The PUFA/SFA ratio is used to inform about the impact of diet or food on cardiovascular health. According to the hypothesis, the higher the value of the index, the better the effect of diet. The analyzed oil samples were divided into three groups. In the first group, where the PUFA/SFA ratio was above 0.5, were two samples (Arbequina and Armonia). Sensation olive oil was characterized by a PUFA/SFA ratio of 0.4. The other three olive oils (Picual, Manzanilla, Cornicabra) were characterized by lower PUFA/SFA ratios, which ranged from 0.25 to 0.31. The heating process in a thin layer caused a decrease in the PUFA/SFA ratio in each of the samples evaluated. However, a much larger decrease was characteristic at 200 °C. The decrease was 2 to 3.8 times greater than when the heating temperature was 170 °C. Higher decreases were characteristic of oils with high PUFA content (Arbequina and Armonia). The higher decrease in PUFA/SFA ratio is associated with a much higher rate of degradation of polyunsaturated fatty acids at 200 °C than at 170 °C. 

Two further indices, the index of atherogenicity (IA) and the index of thrombogenicity (IT), provide us with information on how specific fatty acid groups may exert their effects on the likelihood of increased atherosclerosis, blood clot, and atheroma development and thrombosis. The IA is an indicator of accelerated atherosclerosis, while IT is more indicative of the tendency to form clots in blood vessels and develop cardiovascular disease [44]. According to formulas 1 and 2 presented in the methodological part of the paper, an increase in the values of these indices is associated with a higher proportion of saturated fatty acids (lauric, myristic, palmitic, and stearic). The higher the IA and IT, the higher the analyzed fats are characterized by higher pro-inflammatory properties. Typical and unconventional oils obtained by refining and pressing are characterized by low AI and TI values. Their values can range from 0.05 to 0.29 and 0.05 to 0.56 for AI and TI, respectively [44]. 

For the analyzed olive oil samples, the AI value ranged from 0.15 to 0.18. The TI value ranged from 0.34 to 0.41. In both cases, the value of indices depended on the olive variety used to produce the oil. The lowest values were found for Cornicabra and Picual olive oils, and higher values for Manzanilla and Arbequina. The heating process generally led to an increase in both indices. However, the lower heating temperature (170 °C) induced little change. The value of both indices remained at a similar level compared to unheated oil. The higher process temperature (200 °C) led to more rapid changes, ranging from 2 to 4 times higher compared to 170 °C. 

An increase in the index of atherogenicity and index of thrombogenicity was also observed when heating other oils rich in polyunsaturated fatty acids [45]. However, its increase was most dependent on the fatty acid profile and the proportion of saturated fatty acids. A greater increase in both indices was observed when palm oil was heated than sunflower, rapeseed oil, and olive oil. Heating at high temperatures leads to the degradation of unsaturated fatty acids. Its rate depends on the number of double bonds in the structure of the molecule. Degradation is the fastest in polyunsaturated fatty acids. This is due to the presence of two or more double bonds. In addition, high degradation of PUFA and trace SFA leads to an increase in the % of SFA in the fatty acid pool. A sharp decrease in PUFA and an increase in SFA, especially in oils with a high saturated fraction, contribute to a much faster increase in these indices. 

The HH ratio illustrates the relationship between hypocholesterolemic and hypercholesterolemic fatty acids, which we can observe in oils and foods. The hypocholesterolemic part is PUFA and oleic acid, while the hypercholesterolemic ones are saturated acids from C 12:0 to C 16:0 [43]. Due to the ratio of hypocholesterolemic to hypercholesterolemic fatty acids, a decrease in the value of the index indicates a deterioration in the health-promoting properties of the product. The hypocholesterolemic/hypercholesterolemic ratio of not heated olive oils ranged from 5.42 to 6.61. The lowest value was characteristic for Arbequina olive oil, and the highest for Cornicabra varieties. When heated at 170 °C, the decrease in the H/H ratio was less than 1% of the initial value. When the heating temperature was 200 °C, the decreases were 5 to 10 times higher. The exception was oil of the Manzanilla variety, in which high decreases in the H/H ratio were observed at both temperatures. As before, the changes that were observed during heating were rather typical and did not differ from the literature data. However, changes in indices of lipid nutritional quality during heating of vegetable oils are rarely described in the literature. During heating of vegetable oils, olive oil was one of the least susceptible to changes in nutritional indices. The greatest changes were observed in sunflower and rapeseed oil [45]. In addition, the data presented in the current work indicate that the stability of indices of lipid nutritional quality in olive oil varied depending on the olive variety used for pressing olive oil.

### 2.3. Tocopherols Content 

**Figure 5:**
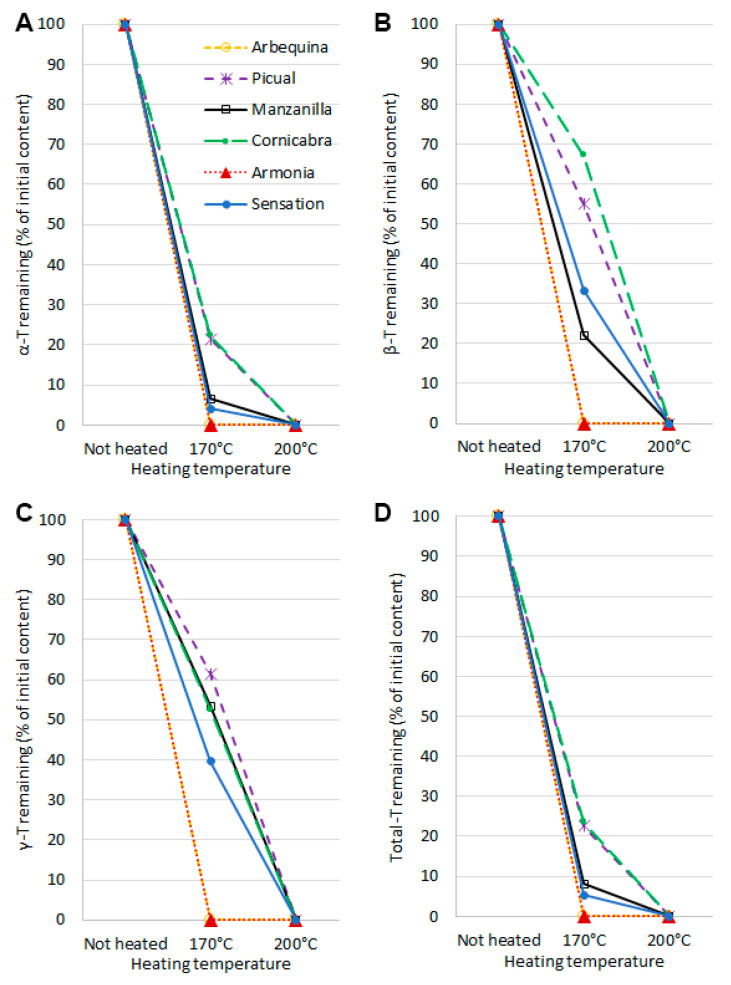
Changes in the content of tocopherols [%] during thin-layer heating olive oils at 170 and 200 °C expressed as residual tocopherols after the heating process. (**A**) α-tocopherol, (**B**) β-tocopherol, (**C**) γ-tocopherol, (**D**) total tocopherols.

The main tocopherols present in oils are α-tocopherol or γ-tocopherols. In the analyzed oils, the tocopherol content ranged from 22.49 mg/100 g of oil (Manzanilla) to 36.16 mg/100 mL of oil (Cornicabra). The tocopherol content of the other olive oils was 28.36, 28.68, 29.00, and 31.75 in Sensation, Arbequina, Armonia, and Picual, respectively. The main tocopherol identified in olive oil was α-tocopherol, which accounted for 95.26 to 97.78% of all tocopherols. When olive oil was heated, drastic decreases in tocopherols were observed. However, the changes were dependent on the type of olive oil and temperature used (Figure 5). At 170 °C, complete or partial degradation of tocopherols was observed. A heating temperature of 200 °C resulted in complete degradation of tocopherols regardless of the type of olive oil. The least stable tocopherol was α-tocopherol. At 170 °C, its complete degradation was observed in Arbequina and Armonia olive oils. In Sensation and Manzanilla olive oils, the loss was 93.5% and 95.99%, respectively. In the other two samples, Cornicabra and Picual, losses were lower at 77.8% and 78.5%, respectively. The other two analyzed tocopherols, β-T and γ-T, had higher stability, however, in only four samples. For Arbequina and Armonia olive oils, heating at 170 °C also completely degraded these tocopherol homologs. For the other four olive oils, the degradation rate varied widely. For β-tocopherol, it ranged from 32.9% in Cornicabra olive oil to 77.9% in Manzanilla olive oil. For γ-tocopherol, it ranged from 38.5% in Picual olive oil to 60.5% in Sensation olive oil. The rapid degradation of α-tocopherol and the higher stability of the other homologs, primarily γ-tocopherol, during heating, is a well-known phenomenon [46]. However, in the case of β-tocopherol, it largely depends on the initial content of tocopherols and the fatty acid profile, as well as the ratio between the surface area in contact with oxygen and the volume of oil used [47]. Analysis of the tocopherol content of unheated olive oils shows that Cornicabra was the oil with the highest content of total tocopherols, as well as their β-T γ-T homologs. Picual also had one of the highest amounts of tocopherols. In the case of Arbequina and Armonia, the tocopherols content in unheated oil was similar to Picual. However, Arbequina and Armonia had the highest PUFA content, more than two times higher, compared to other olive oils. The total degradation of tocopherols in these samples is likely due to their protective role against oxidation processes of polyunsaturated fatty acids.

### 2.4. Total Polar Compounds Content 

Total polar compounds (TPC) content is currently one of the basic indicators of the level of transformations occurring in oils used for frying. It determines the total polar fraction in the oil, native and formed during oxidation, hydrolysis, and thermal processes (polymerization, cyclization, etc.). Its increase indicates the progressive degradation of the oil used. In many countries around the world, exceeding the 24–27% level for TPC indicates the need to replace the oil with a new one [48]. 

**Figure 6:**
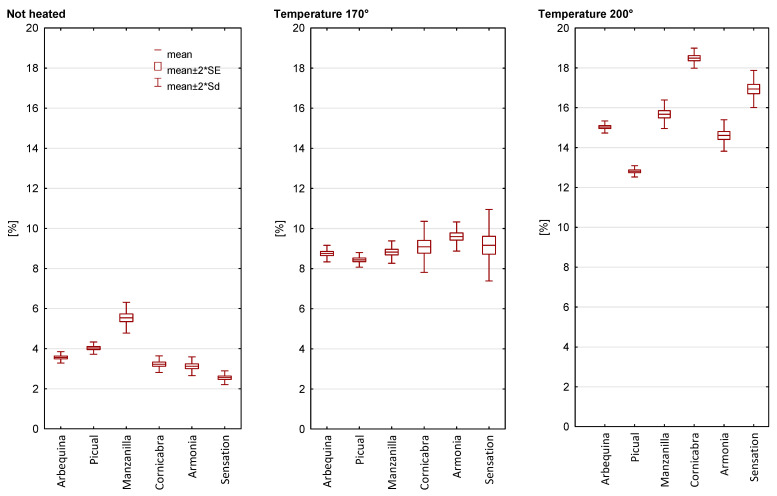
Total polar compounds (TPC) content of unheated and heated olive oil.

The initial content of the polar fraction was at a low level and depended on the oil variety (Figure 6). The TPC content in unheated oils ranged from 2.55% to 5.54%. The content of the polar fraction increased in oils in the following order: Sensation, Armonia, Cornicabra, Arbequina, and Picual. The content of the polar fraction in olive oils can be due to a number of factors, such as the quality and maturity of the raw material, the olive oil extraction technique, and storage conditions. 

The heating process at 170 °C and 200 °C led to an increase in the polar fraction. During heating at 170 °C, the average increase in TPC was 2.6 times. The highest increase was observed in Sensation olive (3.59 times) and the lowest in Manzanilla olive (1.59 times). TPC content after heating ranged from 8.44% (Manzanilla) to 9.60% (Armonia). Increasing the heating temperature to 200 °C led to an increase in the content of the polar fraction by an average of 4.54 times. However, the analyzed olive oils showed greater variation compared to 170 °C. The TPC value in samples heated at a higher temperature ranged from 12.81% (Picual) to 18.49% (Cornicabra). Due to the different initial TPC content, the increase in the content of this fraction ranged from 2.83 to 6.64 times. However, in none of the heated samples did the value of the polar fraction exceed the limit for frying oils. 

The increase in the polar fraction in oils during thermal processes is due to the high temperature of the process and the rapid increase in the rate of chemical reactions occurring in the oil. The increase in temperature or prolongation of the heating process significantly affects the degradation of polyunsaturated fatty acids (accelerating oxidation) but also the degradation of antioxidant compounds (tocopherols or phenolic compounds). [49]. The stability of olive oil depends on the level of antioxidants (tocopherols and phenolic compounds). However, in addition to their total content, an important factor is the content of individual compounds and the mutual ratio between them, as well as the synergism phenomenon. Olive oil produced from different varieties significantly differs in the presence of individual phenolic compounds [50]. In addition, the phenolic compounds present in olive oil vary significantly in antioxidant activity, and the degradation of some occurs faster than others [51]. 

### 2.5. Polimeryztion of Triacyloglycerols 

Triacylglycerol (TAG) polymerization is a process that occurs in heated oils under the influence of high temperature and oxidation. TAG polymers are compounds that, due to their higher molecular weight compared to native TAG, remain in the oil when it is heated. As a result of frying and the process of oil absorption by the fried product, they can become a significant component of the food consumed and negatively affect health. High levels of polymerized fats in the diet can affect the lipid profile of the blood, kidney function, peroxidation of body lipids, and increase oxidative stress on the body [52]. Triacylglycerol polymers also affect the frying process. By increasing viscosity, they impede heat transfer, prolonging the frying process and increasing the amount of fat in the fried food [28,53]. The rate of polymerization depends on many raw materials as well as process factors. The high degree of unsaturation of fatty acids, the presence of pro-oxidants, the low initial quality of oils, as well as high temperature, long time, and developed contact surface with oxygen, accelerate the formation of polymers [29,54,55]. 

Heating olive oil in a thin layer led to an increase in the polar fraction. This fraction included oxidized triacylglycerols, triacylglycerol fragments (diacylglycerols, FFA—free fatty acid), and also triacylglycerol dimers. Dimers of triacylglycerols were characteristic in samples heated at 200 °C (Table 2). At the lower heating temperature, these compounds were observed only in Armonia olive. Their content was 0.79%. The higher process temperature led to the formation of more of these compounds. In samples heated at 200 °C, their content ranged from 1.14% (Picual) to 5.17% (Armonia). The high value of TAG polymers in Armonia oil may be related to the high content of polyunsaturated fatty acids, however, such an effect was not observed in Arbequina oil, which is 70% of Armonia. The samples analyzed, including the olive blends (Armonia and Sensation), were commercially purchased and the Arbequaina and Armonia may be from a different production batch. The data presented in Table 2 show the influence of olive variety on stability during heating. The second important factor affecting degradation transformations, including TAG polymerization, is the temperature of the frying process. Keeping it lower and properly controlled can contribute greatly to reducing the polymerization process. Mixing oils with different fatty acid profiles and lower PUFA content can be another way to reduce the degradation process. The addition of refined coconut oil (RCO) to olive oil allowed the frying sessions of French fries and fish fillets to be extended by half while maintaining similar levels of the polymerized fraction in the not blended olive oil [56]. A similar effect can be observed for Sensation olive (a blend of Arbequina and Picual). Mixing them in the right proportion led to a decrease in the share of PUFA (Arbequina) and an increase in the share of MUFA (Picual). After heating at 200 °C, the level of TAG polymers was low (2.28%).

### 2.6. Principal Component Analysis 

**Figure 7:**
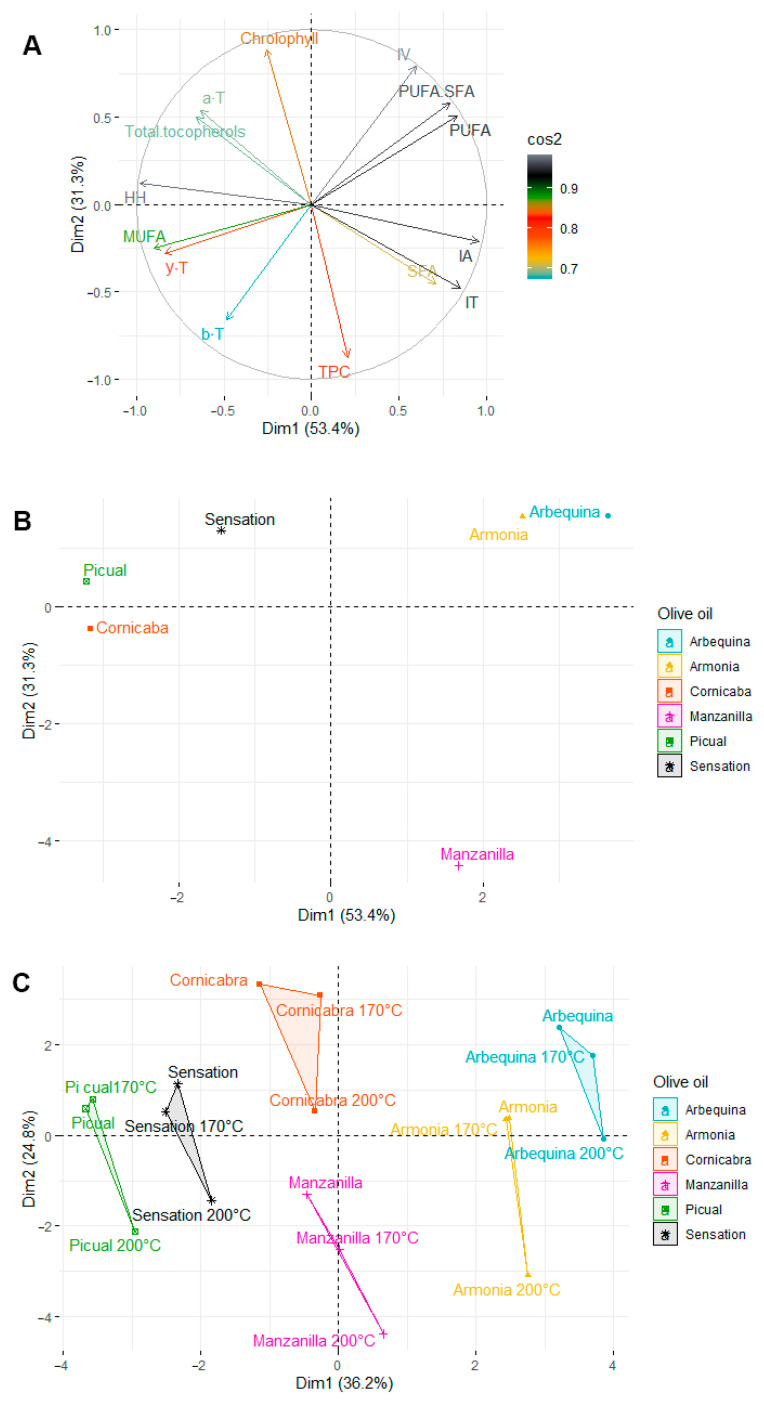
Principal component analysis (PCA) of the loadings plot and the score plot of data from not heated (**A**,**B**) and heated olive oils (**C**).

Principal component analysis (PCA) was the method used to show possible clusters between unheated (Figure 7A,B) and heated olive oils (Figure 7C). For unheated olive oils, the first two principal factors accounted for 84.7% (Dim1 = 53.4% and Dim2 = 31.3%) of the total variation. Factor 1 was mainly positively correlated with the IA value (r = 0.955) and negatively correlated with the HH ratio (r = −0.981) and MUFA content (r = −0.902). Factor 2 was mainly positively correlated with chlorophyll content (r = 0.884) and negatively correlated with TPC content (r = −0.873). The data presented in the score plot (Figure 7B) show a large variation in the analyzed olive oils, which depends mostly on the variety of olives used for pressing the olive oils. 

Arbequina and Armonia are very close to each other, which is due to their great similarity. Armonia is a blend of Arbequina and Picual olive oils (70/30). The second tested blend of Arbequina and Picual (30/70) was Sensation. Due to its high Picual content, it is in closer proximity to this olive oil than to the Arbequina oil. However, not as close as Arbequina and Armonia. This may indicate a stronger influence of Arbequina olive oil on the properties of the resulting blends (Armonia and Sensation) than Picual olive oil. Additionally, in close proximity to Picual is Cornicabra olive oil. However, both are on opposite sides of the *X* axis. Both oils are characterized by a similar proportion of MUFA and also by IA values and HH ratio (the highest of the oils tested). The variation in these oils is due to their chlorophyll content, polar fraction, and tocopherol content. Corniciabra has the highest tocopherol content and a much lower chlorophyll content compared to Picual. The furthest from all the samples is Manzanilla olive oil. Like Arbequina and Armonia, it is on the left side of the *Y* axis and has a similar HH ratio and IA value. Manzanilla had the highest IT value. In addition, it was fundamentally different in terms of tocopherols and chlorophyll content. Both of these parameters were the lowest of the olive oil evaluated.

The heating process led to a large variation in the samples obtained (Figure 7C). Here, too, we observe small distances between the blends of the oils and their main component (Arbequina and Armonia; Picual and Sensation). However, the two pairs are on opposite sides of the *Y*-axis, which is mainly due to the fatty acid profile and the degree of oil degradation. In addition, in the case of these four oils, we observe a small distance between the unheated sample and the sample heated at 170 °C, and a very large distance between these samples and the sample heated at 200 °C. This phenomenon confirms the results obtained about the much smaller effect of 170 °C on the degradation of heated olives. For Cornicabra and Manzanilla, the distances between the unheated and 170 °C heated samples are larger. In addition, comparing the score plot for unheated and heated samples, we observe a shift of these two olives to the central part of the plot.

## 3. Materials and Methods

### 3.1. Materials

The study material consisted of 6 EVOO obtained from Spain. Four samples were single-strain olive oils: Arbequina, Picual, Manzanilla, and Cornicabra. Two samples were a coupage of Arbequina and Picual varieties: Armonia (70% Arbequina and 30% Picual) and Sensation (70% Picual and 30% Arbequina). For research purposes, oil from 2 production batches was obtained. They represented two replicates of the same sample. The single-strain olive oils were packaged in 1 L dark glass bottles. Coupage olive oils were packaged in 3 L metal containers. During research, olive oils were stored in original packaging at 5 °C.

### 3.2. Heating Procedure

Olive oil heating was carried out in a thin layer model. A total of 50 mL of olive oil was heated in a 20 cm diameter pan at 170 ± 5 °C and 200 ± 5 °C. Two different temperatures were chosen to determine the dynamics of changes in olive oil degradation. The heating was conducted in duplicate for each production batch (4 heating processes for one olive oil variety). The heating process was divided into 2 stages. Preheating and heating the sample at the specified temperature. The first stage lasted 7 and 9 min, for temperatures of 170 °C and 200 °C, respectively. The second stage lasted 10 min. Heating was conducted in duplicate for each production batch (4 processes for one oil variety). Heating was conducted on MS-H-Pro (IKA Works, Inc., Wilmington, NC, USA) heating plates with electronic temperature control. During heating, the temperature of the olive oil was monitored using an external thermometer Testo 104 (Testo Sp. z o. o., Pruszków, Poland). After heating, samples were sealed in nitrogen-filled plastic containers and stored at −28 °C until analysis.

### 3.3. Fatty Acid Composition Analysis 

The fatty acid composition was determined according to the AOCS Official Method Ce 1 h-05 [57]. The olive oil samples were dissolved in hexane and transesterified with sodium methylate. The fatty acid methyl esters (FAME) composition was analyzed using an Agilent 7820A GC equipped with a flame ionization detector (FID) (Agilent Technologies, Santa Clara, CA, USA) and SLB-IL111 capillary columns (Supelco, Bellefonte, PA, USA) (100 m, 0.25 mm, 0.20 mm). The GC conditions were as follows: the oven temperature from 150 °C to 200 °C with increasing 1.5 °C/min; the temperature of injector and detector 250 °C, the carrier gas was helium at 1 mL/min; the GC operated in split mode 1:10. FAME composition was identified by comparing the retention times of individual substances with a commercially available standard grain fatty acid methyl ester mix (Supelco, Bellefonte, PA, USA). The results were expressed as a percentage of total fatty acids.

### 3.4. Calculated Iodine Value (CIV) 

The calculated iodine value (CIV) was conducted according to the AOCS Official Method Cd 1c-85 [58] and calculated from fatty acid composition. The method of calculation is based on the percentage of hexadecenoic acid, octadecenoic acid, octadeca-dienoic acid, octadecatrienoic acid, eicosanoid acid, and docosenoic acid.

### 3.5. Indexes of Lipid Nutritional Quality 

#### 3.5.1. Polyunsaturated Fatty Acid/Saturated Fatty Acid (PUFA/SFA)

Polyunsaturated Fatty Acid/Saturated Fatty Acid (PUFA/SFA) is an index normally used to inform about the impact of diet on cardiovascular health [43]. According to the theory, when calculating the index, it is assumed that all PUFA can lower serum LDL cholesterol, while all SFA raise LDL cholesterol. According to the hypothesis, the higher the value of the index, the better the effect of diet. The PUFA/SFA index was also calculated.

#### 3.5.2. Index of Atherogenicity (IA)

The IA index shows the reciprocal relationship between the sum of SFA and the sum of unsaturated fatty acids (UFA) in foods. However, only C12:0, C14:0, and C16:0 acids are considered proatherogenic. Unsaturated fatty acids are considered antiatherogenic because they inhibit atherosclerotic plaque deposition and reduce phospholipid and cholesterol levels [59]. 

The IA index was calculated based on the formula:

IA = [C 12:0 + (4 × C 14:0) + C 16:0]/ΣUFA(1)

#### 3.5.3. Index of Thrombogenicity (IT) 

The IT index characterizes the thrombogenic potential of fatty acid, indicating the tendency to form clots in blood vessels. The index determines the relationship between pro-thrombogenic (SFA) and anti-thrombogenic fatty acids (MUFA, n-3, and n-6 PUFA) [59]. 

The IA index was calculated based on the formula:

IT = (C 14:0 + C 16:0 + C 18:0)/[(0.5 × ΣMUFA) + (0.5 × Σn − 6 PUFA) + (3 × Σn − 3 PUFA) + (n − 3/n − 6)](2)

#### 3.5.4. Hypocholesterolemic/Hypercholesterolemic (HH) Ratio

The HH ratio illustrates the relationship between hypocholesterolemic and hypercholesterolemic fatty acids. Mainly, it is the relationship between sum of oleic acid (C 18:1) and polyunsaturated fatty acids (hypocholesterolemic part), and saturated fatty acids from C 12:0 to C 16:0 (hypercholesterolemic part) [43].

The HH ratio index was calculated based on the formula:

HH = (cis − C 18:1 + ΣPUFA)/(C 12:0 + C 14:0 + C 16:0)(3)

### 3.6. Chlorophyll Content Analysis

The chlorophyll content was determined according to the AOCS Official Method Cc 13i-96 [60]. The chlorophyll pigments were determined by measuring the absorbance at 670 nm, correcting the result for the background absorption and calculating the content. The results are expressed as mg of pheophytin a in 1 kg of oil.

### 3.7. Tocochromanols Analysis 

The tocopherols content was determined according to Siger et al. [61]. The tocopherols content was analyzed using a Waters HPLC system (Waters, Milford, MA, USA) equipped with a photodiode array detector (Waters 2998 PDA), a fluorimetric detector (Waters 474), and a LiChrosorb Si 60 column (250 × 4.6 mm, 5 µm, Merck, Darmstadt, Germany). The HPLC conditions were as follows: mobile phase was a mixture of n-hexane with 1.4-dioxane (96:4, *v*:*v*); the flow rate was 1.0 mL/min; injection sample volume was 10 mL; the excitation wavelength was set at λ = 295 nm and the emission wavelength at ʎ = 330 nm. The tocopherols were identified by comparison with retention times of standards purchased from Merck (>95% of purity).

### 3.8. Total Polar Compounds (TPC) Analysis 

Total polar compounds in the olive oil were determined according to the AOCS Official Method 982.27 [62]. The sample was dissolved in toluene and applied to the top of a column packed with silica gel (silica gel 60, 63–200 µm, Sigma-Aldrich, Poznan, Poland). A mixture of hexane and diisopropyl ether (82:18, *v*:*v*) was used to elute a nonpolar fraction of olive oil. After evaporation of the solvent, the nonpolar fraction was weighed. From the mass difference between the oil sample and nonpolar fraction, the polar fraction was calculated. The results were expressed as % of the content of oil.

### 3.9. Polymerized Triacylglycerols (PTAG) Analysis 

The composition of triacylglycerol (TAG) polymers in heated oil was determined according to the AOCS Official Method 993.25 [63]. The composition of TAG polymers was analyzed using an Infinity 1290 HPLC (Agilent Technologies, Santa Clara, CA, USA) equipped with ELSD (Evaporative Light Scattering Detector) and two Phenogel columns (100 Å and 500 Å, 300 × 7.8 mm) (Phenomenex, Torrance, CA, USA). The HPLC conditions were as follows: flow rate of dichloromethane (DCM) was 1 mL/min; column and detector temperature: 30 °C; detector pressure 2.5 bars; injection volume of sample 1 mL. 

### 3.10. Statistical Analysis 

All assays were replicated four times. Mean values and standard deviations were calculated with Microsoft Office Excel 2019 (Microsoft Corporation, Redmond, WA, USA). STATISTICA 13.3 (Dell Software Inc., Round Rock, TX, USA) was used to calculate standard errors and significant differences between means (*p* < 0.05, analysis of variance ANOVA), Tukey’s multiple range test. R studio 2023.03.1 +446with packages FactoMineR v2.4 and factoextra v1.0.7 was the software used for principal components analysis (PCA).

## 4. Conclusions

Olive oil is commonly used for frying processes. However, the effect of high temperature leads to a change in its quality and degradation of its components. As a result of the analysis, it was found that the quality and susceptibility to degradation depended on the olive variety and the temperature of the heating process. The analyzed oils were characterized by high initial quality, but they varied and depended on the olive variety. The main differentiating factor was the fatty acid profile and share of PUFA, as well as the presence of tocopherols. The share of PUFA ranged from 3.95% (Picual) to 10.53% (Arbequina). For other olive oils, it was 5.34%, 5.48%, 6.73%, and 9.53% for Cornicabra, Manzanilla, Sensetion, and Armonia, respectively. The tocopherol content ranged from 22.49 mg/1 g of oils (Manzanilla) to 36.16 mg/1 g of oils (Cornicabra). Due to the fatty acid profile, olive oils are characterized by different values of indexes of lipid nutritional quality. Due to the nutritional quality of the olive oils, they can be arranged in the order Cornicabra > Picual > Sensation > Armonia > Arbequina > Manzanilla. Heating the oil led to deterioration. However, the degree of degradation depended on the temperature and type of olive oil. At 170 °C, the oils were characterized by higher stability compared to 200 °C. Virtually no polymerization of triacylglycerols was observed and less loss of tocopherols was observed. After heating, complete degradation of tocopherols was observed in Arbequina and Armonia samples. In Sensation and Manzanilla, losses ranged from 93 to 95%. In the other two olive oils, Picual and Corniciabra, the tocopherol content was 22.5% and 23.5% of the initial level, respectively. The most stable homologs in the oils tested were γ- and β-tocopherol. Increasing the heating temperature to 200 °C caused significant and rapid degradation changes, confirmed by the presence of polymerization of triacylglycerols and complete degradation of tocopherols in all samples. The least triacylglycerol polymers were observed in Picual olive oil. Armonia olive oil had the highest content of TAG polymers.

## Figures and Tables

**Table 1:** Characteristics of unheated olive oils.

	Arbequina	Picual	Manzanilla	Cornicabra	Armonia	Sensation
Fatty acid composition [%]						
C 16:0	15.61 ± 0.16 ^c^	13.00 ± 0.07 ^a^	14.58 ± 0.28 ^b^	13.19 ± 0.14 ^a^	14.76 ± 0.08 ^b^	13.60 ± 0.13 ^a^
C 16:1	1.28 ± 0.06 ^b^	0.91 ± 0.01 ^a^	1.26 ± 0.06 ^b^	1.02 ± 0.04 ^a^	1.18 ± 0.01 ^bc^	1.04 ± 0.03 ^ac^
C 18:0	2.10 ± 0.04 ^a^	2.17 ± 0.03 ^a^	3.22 ± 0.04 ^b^	3.58 ± 0.10 ^c^	2.07 ± 0.03 ^a^	2.11 ± 0.06 ^a^
C 18:1	69.49 ± 0.45 ^c^	79.02 ± 0.07 ^d^	74.38 ± 0.30 ^a^	75.73 ± 0.17 ^b^	71.65 ± 0.13 ^d^	75.53 ± 0.47 ^ab^
C 18:2	9.89 ± 0.07 ^e^	3.12 ± 0.06 ^b^	4.75 ± 0.08 ^a^	4.49 ± 0.06 ^a^	8.90 ± 0.07 ^d^	5.99 ± 0.07 ^c^
C 18:3	0.64 ± 0.04 ^ab^	0.83 ± 0.01 ^bc^	0.73 ± 0.04 ^abc^	0.85 ± 0.08 ^c^	0.63 ± 0.03 ^a^	0.74 ± 0.06 ^abc^
C 22:0	0.41 ± 0.03 ^a^	0.37 ± 0.04 ^a^	0.51 ± 0.03 ^ab^	0.56 ± 0.04 ^b^	0.41 ± 0.04 ^a^	0.42 ± 0.03 ^ab^
C 20:1	0.32 ± 0.03 ^a^	0.27 ± 0.03 ^a^	0.28 ± 0.01 ^a^	0.27 ± 0.01 ^a^	0.13 ± 0.00 ^b^	0.29 ± 0.04 ^a^
C 22:0	0.12 ± 0.00 ^a^	0.12 ± 0.03 ^a^	0.14 ± 0.03 ^ab^	0.15 ± 0.01 ^b^	0.10 ± 0.01 ^a^	0.12 ± 0.01 ^ab^
C 22:1	0.08 ± 0.01 ^a^	0.12 ± 0.03 ^a^	0.09 ± 0.03 ^a^	0.09 ± 0.03 ^a^	0.10 ± 0.00 ^a^	0.10 ± 0.03 ^a^
C 24:0	0.06 ± 0.01 ^a^	0.07 ± 0.01 ^a^	0.06 ± 0.00 ^a^	0.07 ± 0.00 ^a^	0.07 ± 0.01 ^a^	0.06 ± 0.01 ^a^
SFA	18.30 ± 0.24 ^cd^	15.73 ± 0.04 ^a^	18.51 ± 0.24 ^d^	17.55 ± 0.28 ^bc^	17.41 ± 0.04 ^b^	16.31 ± 0.24 ^a^
MUFA	71.17 ± 0.35 ^c^	80.32 ± 0.00 ^e^	76.01 ± 0.28 ^a^	77.11 ± 0.25 ^b^	73.06 ± 0.14 ^d^	76.96 ± 0.37 ^ab^
PUFA	10.53 ± 0.11 ^e^	3.95 ± 0.04 ^b^	5.48 ± 0.04 ^a^	5.34 ± 0.03 ^a^	9.53 ± 0.10 ^d^	6.73 ± 0.13 ^c^
ω6/ω3 ratio	15.48 ± 0.92 ^e^	3.76 ± 0.13 ^a^	6.52 ± 0.5 ^c^	5.31 ± 0.6 ^b^	14.14 ± 0.52 ^e^	8.11 ± 0.52 ^d^
CIV	80.09 ± 0.07 ^b^	76.56 ± 0.07 ^a^	75.59 ± 0.20 ^c^	76.37 ± 0.34 ^a^	79.98 ± 0.07 ^b^	78.55 ± 0.05 ^d^
Tocopherols [mg/1 g of oils]						
α-T	27.73 ± 0.60 ^a^	30.85 ± 0.64 ^c^	21.43 ± 0.30 ^b^	34.85 ± 0.32 ^d^	28.27 ± 0.58 ^a^	27.68 ± 0.69 ^a^
β-T	0.16 ± 0.05 ^a^	0.27 ± 0.07 ^ab^	0.48 ± 0.10 ^c^	0.50 ± 0.07 ^c^	0.25 ± 0.05 ^ab^	0.40 ± 0.07 ^bc^
γ-T	0.47 ± 0.09 ^a^	0.64 ± 0.11 ^ab^	0.59 ± 0.03 ^a^	0.82 ± 0.11 ^b^	0.49 ± 0.09 ^a^	0.60 ± 0.06 ^a^
Total T	28.36 ± 0.51 ^a^	31.75 ± 0.50 ^c^	22.49 ± 0.38 ^b^	36.16 ± 0.31 ^d^	29.00 ± 0.64 ^a^	28.68 ± 0.74 ^a^
Chlorophyll [1 mg phenophytin/1 kg of oil]						
	17.30 ± 0.25 ^a^	20.27 ± 0.36 ^b^	9.51 ± 0.28 ^c^	14.30 ± 0.41 ^d^	17.79 ± 0.59 ^a^	19.89 ± 0.45 ^b^
Total Polar Compounds [%]						
	3.57 ± 0.14 ^ab^	4.03 ± 0.15 ^b^	5.54 ± 0.38 ^d^	3.23 ± 0.21 ^a^	3.13 ± 0.23 ^a^	2.55 ± 0.17 ^c^

Values are means of four determinations ± SD. Means in the same row, followed by different small letters, indicate significant differences (*p* < 0.05) between samples. α-T—α-tocopherol, β-T—β-tocopherol, γ-T—γ-tocopherol, SFA—saturated fatty acid, MUFA—monounsaturated fatty acid, PUFA—polyunsaturated fatty acid, CIV—Calculated Iodine Value.

**Table 2:** Changes of dimer of triacylglycerols (TAG) content [%] in olive oils.

Samples	Arbequina	Picual	Manzanilla	Cornicabra	Armonia	Sensation
Not heated	-	-	-	-	-	-
170 °C	-	-	-	-	0.79 ± 0.03	-
200 °C	2.06 ± 0.14 ^b^	1.14 ± 0.1 ^a^	3.24 ± 0.25 ^d^	2.14 ± 0.12 ^b^	5.17 ± 0.23 ^e^	2.28 ± 0.10 ^c^

Values are means of four determinations ± SD. Means in the same row, followed by different small letters, indicate significant differences (*p* < 0.05) between samples.

## Data Availability

All data generated or analyzed during this study are included in this published article.
